# Plasma Biomarkers of Brain Atrophy in Alzheimer's Disease

**DOI:** 10.1371/journal.pone.0028527

**Published:** 2011-12-21

**Authors:** Madhav Thambisetty, Andrew Simmons, Abdul Hye, James Campbell, Eric Westman, Yi Zhang, Lars-Olof Wahlund, Anna Kinsey, Mirsada Causevic, Richard Killick, Iwona Kloszewska, Patrizia Mecocci, Hilkka Soininen, Magda Tsolaki, Bruno Vellas, Christian Spenger, Simon Lovestone, for the AddNeuroMed consortium

**Affiliations:** 1 Laboratory of Behavioral Neuroscience, National Institute on Aging, National Institutes of Health, Baltimore, Maryland, United States of America; 2 King's College London, Institute of Psychiatry, London, United Kingdom; 3 Proteome Sciences plc, Coveham House, Cobham, United Kingdom; 4 Department of Clinical Science, Intervention and Technology, Division of Radiology, Karolinska Institutet, Stockholm, Sweden; 5 Department of Neurobiology, Care Sciences and Society, Section of Clinical Geriatrics, Karolinska Institutet, Stockholm, Sweden; 6 Department of Old Age Psychiatry and Psychotic Disorders, Medical University of Lodz, Lodz, Poland; 7 Section of Gerontology and Geriatrics, Department of Clinical and Experimental Medicine, University of Perugia, Perugia, Italy; 8 Department of Neurology, University of Eastern Finland and University Hospital, Kuopio, Finland; 9 Department of Neurology, Aristotle University of Thessaloniki, Thessaloniki, Greece; 10 Department of Internal and Geriatrics Medicine, Hôpitaux de Toulouse, Toulouse, France; McGill University/Douglas Mental Health University Institute, Canada

## Abstract

Peripheral biomarkers of Alzheimer's disease (AD) reflecting early neuropathological change are critical to the development of treatments for this condition. The most widely used indicator of AD pathology in life at present is neuroimaging evidence of brain atrophy. We therefore performed a proteomic analysis of plasma to derive biomarkers associated with brain atrophy in AD. Using gel based proteomics we previously identified seven plasma proteins that were significantly associated with hippocampal volume in a combined cohort of subjects with AD (N = 27) and MCI (N = 17). In the current report, we validated this finding in a large independent cohort of AD (N = 79), MCI (N = 88) and control (N = 95) subjects using alternative complementary methods—quantitative immunoassays for protein concentrations and estimation of pathology by whole brain volume. We confirmed that plasma concentrations of five proteins, together with age and sex, explained more than 35% of variance in whole brain volume in AD patients. These proteins are complement components C3 and C3a, complement factor-I, γ-fibrinogen and alpha-1-microglobulin. Our findings suggest that these plasma proteins are strong predictors of *in vivo* AD pathology. Moreover, these proteins are involved in complement activation and coagulation, providing further evidence for an intrinsic role of these pathways in AD pathogenesis.

## Introduction

There is an urgent need for biomarkers of Alzheimer's disease (AD); especially to detect the early stages of disease. Such biomarkers have considerable potential in both clinical practice and research where they may accelerate the development of novel disease-modifying treatments [1]. In both the United States and Europe public/private consortia are conducting trials to discover such biomarkers [2], [3]. Strategies for biomarker discovery in AD are well advanced using neuroimaging and assays of candidate proteins in cerebrospinal fluid (CSF). However these methods may not be widely available for use in large, community based, multicentre studies or in the routine clinical care of large numbers of frail elderly people.

Approaches to biomarker discovery in AD have traditionally focused on demonstrating the power of candidate biomarkers to discriminate between cases and controls and have therefore relied upon standard sensitivity and specificity measures to evaluate the clinical utility of such biomarkers. We have previously used this strategy in a large proteomic analysis of plasma to derive a panel of proteins differentiating AD from age-matched healthy control subjects [4]. Employing two dimensional gel electrophoresis (2DGE) followed by liquid chromatography tandem mass spectrometry (LC/MS/MS), we identified 15 plasma proteins whose concentrations were significantly different in AD compared to control subjects. Using semi-quantitative Western blotting, we subsequently validated two proteins; complement factor-H (CFH) and alpha2-macroglobulin (A2M) as AD-specific plasma biomarkers.

Although the above standard approach relying upon the binary distinction of differentiating disease from control may be useful, it may not be suitable for the identification of biomarkers accurately reflecting or measuring *in vivo* disease pathology in subjects with early or established AD. This attribute is in turn a key criterion for an AD biomarker [5] and one that might be especially useful in the setting of clinical trials for the enrichment of patient populations with varying severities of disease pathology. Case versus control approaches to biomarker discovery in AD also ignore the considerable overlap of pathologies such as those underlying vascular injury which are commonly observed in post mortem studies of AD patients [6]. An alternative approach is to therefore seek novel markers based primarily on their association with established metrics of disease pathology. We have successfully used this approach recently to identify plasma clusterin concentration as a marker of pathology in AD [7]. In the current study, we report the validation of a panel of plasma proteins associated with brain atrophy in AD.

## Methods

### Subjects and samples

We recruited 262 subjects (AD, N = 79); MCI, N = 88; and control N = 95) as part of AddNeuroMed, a multi-centre European study for the identification of AD biomarkers. Assessment, imaging and diagnostic procedures have been previously reported [8] [2].

### Ethics committee approval

This study was approved by the South London and Maudsley NHS Foundation Trust ethics committee. Ethics committee approval was also obtained at each of the participating centres in accordance with the Alzheimer's Association's published recommendations [9].

### MRI Data Acquisition

The primary outcome measure for validation was whole brain volume; chosen as an *in vivo* measure of pathology [10], [11]. Whole-brain sagittal three-dimensional MP-RAGE images (TR = 8.6, TE = 3.8, 256×192 acquisition matrix, 180×1.2 mm slices) were obtained from all subjects on a 1.5T MR system at each of the 6 participating centres. Whole brain volumes, consisting of grey and white matter with CSF excluded and normalised to intracranial volume, were determined using an artificial neural network classifier [12]. Quality control of the MR systems was performed using the ADNI test object [13] and comparability between centres assured by repeat scanning of two volunteers on each system (whole brain volume coefficient of variation = 1.7%).

### Selection of candidate biomarkers associated with brain atrophy

The selection of candidate plasma proteins for quantitative immunoassays in this report was based upon an earlier discovery-phase study in a separate cohort of AD (N = 27) and MCI (N = 17) subjects that identified the concentrations of seven plasma proteins as being significantly associated with hippocampal volume. These seven proteins were complement C3, γ-fibrinogen (Fibrinogen gamma chain), serum albumin, complement factor-I (CFI), clusterin, α1-microglobulin, and serum amyloid-P (SAP). The detailed description of these discovery-phase 2DGE and LC/MS/MS experiments has been previously reported [7]. Briefly, the discovery-phase studies used optical densities of silver-stained protein spots in 2DGE gels and examined their association with hippocampal volumes estimated by manual tracing. Of these seven proteins, we recently validated plasma clusterin concentration as a candidate AD biomarker by reporting its association with disease severity, pathology and progression [7]. In the present report, our main aim was to examine the association with AD pathology of all the other plasma proteins (except clusterin and albumin) identified in our previous discovery-phase study. We therefore selected complement C3 and its cleavage product C3a, γ-fibrinogen, complement factor-I (CFI), α1-microglobulin, and serum amyloid-P (SAP) for validation in the current report using alternative methods in a large independent cohort of AD (N = 79), MCI (N = 88) and control (N = 95) subjects. We employed quantitative immunoassays to measure protein concentrations and automated estimates of whole brain volume using MRI images for measurement of brain atrophy.

### Immunoassays

We used ELISA-based immunoassays where available (C3, C3a, and α-1-microglobulin) and semi-quantitative Western blotting in the remainder (CFI, SAP, γ-fibrinogen) (table S1 and table S2). All samples were run in quadruplicate except α-1-microglobulin which was run in duplicate. For Western blots, a reference plasma sample (consisting of at least 15 combined plasma samples from individuals collected in different centres) was run in duplicate on every gel and signals for CFI, SAP and γ-fibrinogen were normalised to the mean value of this sample.

### Statistics

Inter-group differences in age, sex and education were tested by univariate general linear models. Differences in neuroimaging measures, MMSE and plasma concentrations of candidate biomarkers were tested by univariate general linear models after covarying for age. In order to account for the effects of age and sex, we first included these two variables alone as predictors of variance in whole brain volume in each of the AD, MCI and control groups using partial least squares (PLS) regression. Subsequently, the plasma protein concentrations of the candidate biomarkers were scaled to unit variance and together with age and sex, were entered into PLS regression analyses to derive models predictive of whole brain volume in each group (unit variance scaling gives both high and low variance variables equal importance in the model). In exploratory analyses, education was included as a covariate in all the PLS regression models. As it was found not to contribute to variance in whole brain volume, it was excluded from the final optimal PLS model.

The predictive ability of the PLS model was assessed using a seven-fold cross validation procedure and summarised as the root mean square error of prediction (RMSEP): 
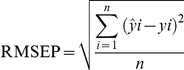
where *ŷ_i_*-*y_i_* represents the residuals between predicted and actual values of whole brain volume. The RMSEP is analagous to a standard deviation of the differences between predicted and actual values of whole brain volume.

## Results

We have previously reported the gel-based proteomics discovery of candidate plasma proteins associated with hippocampal atrophy in AD [7]. Having previously identified a set of proteins associated with hippocampal volume in disease state, we set out, in the current study, to validate these findings using alternative methods in a larger, independent cohort of subjects. We established, *a priori*, outcome criteria for validation; the primary outcome being association with whole brain volume, chosen both as an excellent discriminator of disease state [10], [11] and a measure of atrophy readily suitable for analysis of MRI data obtained from a multi-centre study with fewer problems of rater-variability than manual estimates of hippocampal volume. Secondary outcomes were differences in concentrations of markers between diagnostic groups and/or correlation with clinical measures of disease severity (MMSE for cognition and Clinical Dementia Rating for global severity).

### Subject characteristics

Patients with AD (N = 79; 75.6±6.0 years) were slightly older than both MCI (N = 88; 74.6±7.0 years; non-significant) and control subjects (N = 95; 73.1±6.7 years; *p* = 0.005; LSD post-hoc test). There were no significant differences in gender between the groups. Whole brain volume was significantly decreased in the AD group compared to both control (*p*<0.001) and MCI (*p*<0.001) subjects (table 1). Table 2 shows the mean plasma concentrations of the assayed proteins with the corresponding standard errors.

**Table 1:** Sample characteristics of AD, MCI and control participants in this study.

	AD (n = 79)	MCI (n = 88)	Control (n = 95)
Sex (M/F)	28/51	42/46	43/52
Age (years)	76.0 (6.0)*	74.6 (5.9)	73.1 (7.0)
Education (years)	7.9 (4.0)†	9.2 (4.3)††	10.8 (4.8)
Disease duration (years)	3.9 (2.4)		
MMSE	20.9 (4.6)§	27.3 (1.6)§§	29 (1.2)
Whole brain volume normalised to total ICV.	0.82 (0.03)¶ ^,^ ¶¶	0.85 (0.03)	0.86 (0.030)

Values are expressed as mean ± (SD).

*Differs from control; *p* = 0.007.

§ Differs from control; p<0.001.

§§ Differs from control; *p*<0.001.

¶ differs from control; *p*<0.001.

¶¶ differs from MCI; *p*<0.001.

† Differs from control; p<0.001.

†† Differs from control; p<0.02.

**Table 2:** Plasma concentrations of assayed candidate biomarkers with their corresponding standard errors.

	AD	MCI	Control
C3 (µg/µl)	1588.0 (170.7)	1282.1 (110.5)	1167.1 (65.2)
C3a (ng/ml)	2653.3 (134.5)	2629.9 (136.2)	3064.0 (118.4)
A1M (mg/l)	16.7 (0.94)	17.27 (0.93)	15.58 (1.0)
CFI*	0.86 (0.01)	0.86 (0.01)	0.88 (0.01)
Gamma-fibrinogen*	0.92 (0.01)	0.96 (0.01)	0.94 (0.01)
SAP*	1.12 (0.05)	1.1 (0.04)	1.12 (0.05)

CFI, Gamma fibrinogen and SAP were assayed by Western blotting and their concentrations are in arbitrary units of optical density*.

### Partial least squares regression of whole brain volume against predictor variables

**Figure 1:**
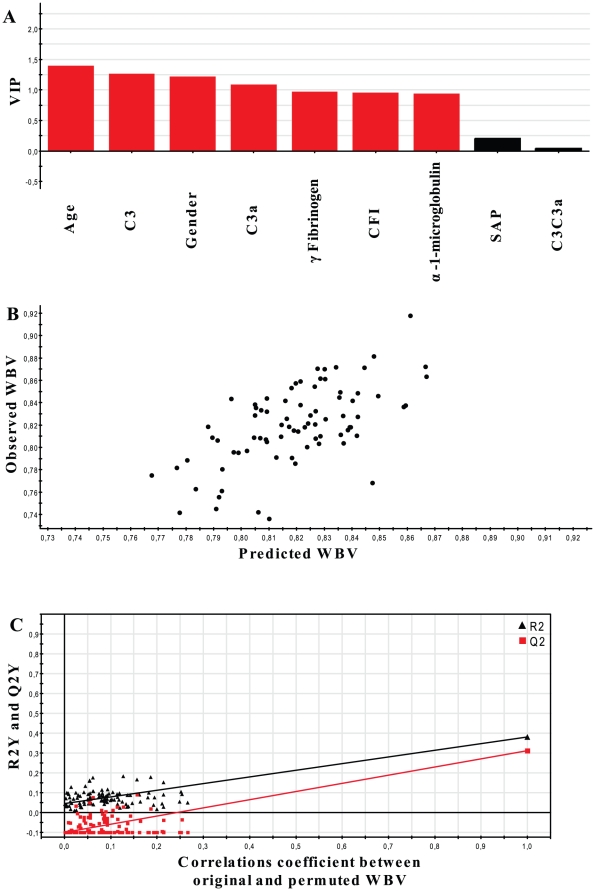
Plasma proteins associated with whole brain volume in Alzheimer's disease. **A**. Variable influence on projection (VIP) plot summarising the overall contribution of each predictor variable to the PLS model for brain volume in AD, summed over all components and weighted according to the Y variation accounted for by each component. Black bars represent variables contributing the least (SAP and C3∶C3a) to variance in the brain volume and therefore eliminated in the final PLS model. **B**. The result of a seven-round cross validation exercise in which every point represents test data not used in the model-building. Plots of observed versus predicted values of normalised whole brain volume (WBV) in AD patients using a single-component PLS model constituted by age, sex, C3a, C3, γ-fibrinogen, α-1-microglobulin and CFI (regression line is represented by the equation: observed value = [1.00±0.126×predicted value]+0.0004±0.102; root mean square error of predictions = 0.027). **C**. Internal validation of the final PLS model predicting whole brain volume in AD demonstrating clear decreases in model performance as the whole brain volume data are permuted relative to the predictor variables. R2Y (black triangles) describes how well the derived model fits the data and is the proportion of the sum of squares explained by the model. Q2 (red squares) describes the predictive ability of the derived model and is the cross validated R2Y. The pair of R2 and Q2 values at the extreme right represent the optimal PLS model constituted by age, sex, C3a, C3, γ-fibrinogen, α-1-microglobulin and CFI. The cluster of R2 and Q2 values at the left represent the PLS models derived by permutating the whole brain volume data relative to the predictor variables and show a clear decline in performance.

In initial exploratory analyses, we first examined unadjusted univariate associations between concentrations of the six plasma proteins and whole brain volume in the AD group (table 3). Age and sex together accounted for 19.7% of variance in whole brain volume in the AD group. Single component PLS models were then fitted to whole brain volume wherein the predictor variables included age, sex, concentrations of the six plasma proteins and the ratio of complement C3∶C3a. The latter measure was included as a predictor variable as it is an accepted marker of complement activation [14]. The model explaining the greatest variance in whole brain volume was in the AD group, where a single-component PLS model explained 37.7% of the variance (R2Y) in brain volume (Q2 provides an estimate of how well the model predicts the Y data and R2X denotes variance explained in the predictor variables) (table 2). A further refinement of this model was achieved by eliminating those predictor variables contributing the least to explaining variance in whole brain volume. Inspection of the variable influence on projection (VIP) plot showed that the ratio of C3∶C3a and SAP concentration contributed least to explaining variance in whole brain volume in AD and these variables were therefore eliminated (figure 1A). This refinement led to deriving a final optimal single-component PLS model with age, sex, complement C3, C3a, γ-fibrinogen, α-1-microglobulin and CFI that could together explain 38.2% of variance in whole brain volume in subjects with AD (table 4).

**Table 3:** Univariate associations between plasma concentrations of assayed candidate biomarkers and whole brain volume in AD; R = Pearson correlation coefficient; p = 2-tailed statistical significance.

Plasma protein	R/p
C3	0.31/0.006
C3a	0.27/0.02
A1M	−0.23/0.04
CFI	0.24/0.04
Gamma-fibrinogen	0.24/0.03
SAP	0.05/0.65

**Table 4:** Summary of the partial least squares (PLS) models fitted to whole brain volume in AD; R2X-variance explained in the predictor variables; R2Y-variance explained in the response variable i.e. whole brain volume; Q2-goodness of prediction of the PLS model.

Number of components	Predictor variables	R2X	R2Y	Q2
1	Age, Sex	0.53	0.197	0.187
1	Age, Sex, C3, C3a, C3∶C3a, CFI, SAP, γ-fibrinogen, α1-microglobulin	0.186	0.377	0.295
1*	Age, Sex, C3, C3a, CFI, γ-fibrinogen, α1-microglobulin	0.277	0.382	0.311

*Denotes final optimal PLS model, after eliminating those variables contributing the least to explaining variance in whole brain volume.

### Cross validation of PLS model of plasma proteins predicting brain volume

The final PLS model for whole brain volume in AD consisting of age, sex, C3a, C3, γ-fibrinogen, α-1-microglobulin and CFI gave a low RMSEP value of 0.027 indicating good predictive power (figure 1B).

### Internal validation of the PLS model for whole brain volume in AD

Further ‘internal’ model validation was effected by randomising the positions of the Y data in relation to their corresponding rows in the X matrix (typically 100 separate row permutations were performed) and observing the effect of this randomisation on the R2Y and Q2 values. Randomisation of the Y data considerably reduced R2Y and Q2 (figure 1C) in comparison to the original model, thereby indicating its validity. Furthermore, the results of this response permutation testing suggest that the likelihood of deriving a model with comparable predictive ability purely by chance was less than 1%, further indicating the robustness of the PLS model for whole brain volume in AD.

### Clinical correlations with plasma biomarkers

We also examined the plasma concentrations of these proteins in relation to diagnosis and clinical measures of severity as secondary outcomes. Plasma C3 was significantly elevated in AD (*p* = 0.03) patients relative to controls. We also observed a trend for association between plasma C3 concentration and MMSE score in the combined group of AD and MCI subjects (r = −0.14, p = 0.07). Plasma γ-fibrinogen was significantly increased in MCI subjects versus AD (*p* = 0.03).

## Discussion

We have adopted a novel approach to the discovery of biologically relevant plasma biomarkers in early AD. Our aim was to identify peripheral markers of AD by their association with established neuroimaging measures of pathology and then to validate these by alternative quantitative methods in a large and independent test population.

Multiple lines of evidence suggest that peripheral fluids such as plasma may be a rich source of biomarkers in AD. Such markers might reflect a systemic metabolic signature of AD or be a change in plasma secondary to a disease-specific process in the brain [15]. We have previously used a proteomic approach to identify plasma proteins differentially expressed in established AD [4]. Others have used arrays of candidate proteins, finding remarkably high sensitivity and specificity for diagnosis of established AD versus controls [16]. Both candidate and ‘data-driven’ approaches (proteomics, transcriptomics etc) tend to use disease status, either case versus control or control/MCI progression to case, as the primary outcome variable in discovery studies. Where the discovery paradigm uses large scale or array-based technologies, this can result in the identification of potential biomarkers with no known, or at best, uncertain, involvement in disease. Furthermore this binary distinction (disease/no disease) may result in the discovery of biomarkers that show excellent diagnostic or predictive characteristics but lack sensitivity in relation to disease progression or severity. To avoid these problems, we sought to discover, using proteomics, biomarkers where the primary outcomes were associations with well-established neuroimaging measures of disease pathology.

In the discovery-phase proteomics study which led us to the candidate plasma proteins of interest in the current report, we used hippocampal atrophy as a measure of disease pathology [7]. For validation of candidate markers in the present study, we chose an alternative measure of brain atrophy to overcome some of the limitations of manual hippocampal volumetry. The chief advantage of whole brain over manual estimates of hippocampal volume is that the automated calculation of whole brain volume is not subject to inter-rater variability and is therefore readily utilisable in large multi-centre studies such as ours.

Moreover, like hippocampal atrophy, whole brain atrophy is also an early event in the disease, an excellent discriminator of disease state and correlates closely with longitudinal measures of atrophy [10], [11], [17]. Automated cross-sectional measurements of normalised whole brain volume have also been compared with longitudinal measures of rates of whole brain atrophy [11]. These studies have reported that the cross-sectional measurement of whole brain volume is nearly as powerful a discriminant between AD patients and controls as longitudinal observations on rates of whole brain atrophy. Equally importantly, there is a highly significant correlation between cross-sectional and longitudinal measures of whole brain atrophy in AD. The latter has also been used as a neuroimaging biomarker in a clinical trial of AD [18]. Cross-sectional measurement of whole brain volume was recently shown to differentiate between MCI subjects progressing to AD and those that remained stable [10]. In subjects with MCI and established AD, there was also a highly significant association between baseline whole brain volume and CSF Aβ_1–42_, levels, further suggesting that this neuroimaging measure reflects an integral feature of AD neuropathology [19].

Our previous discovery-phase study demonstrated that seven plasma proteins were significantly associated with hippocampal volume in a combined cohort of AD and MCI subjects [7]. In the current report, we confirmed a significant association between these plasma proteins and whole brain volume in AD. Five proteins from the original panel of candidate biomarkers explained 18% of variance in brain volume in the AD group. Together with age and sex, these proteins could explain more than 35% of variance in brain volume in AD patients. Further cross validation and response permutation testing confirmed a robust predictive power of this PLS model for whole brain volume in AD.

Our results demonstrate that we have identified a panel of plasma proteins that are predictors of current disease severity as measured by well-established neuroimaging markers of pathology. Moreover, their association with core neuropathological features of AD suggests that these proteins are not merely non-specific markers of disability in the elderly, but biologically relevant proteins accurately reflecting disease pathology.

Most of the plasma proteins associated with neuroimaging measures of disease pathology in this study are components or regulators of the complement system and coagulation pathway. Multiple lines of evidence support a role for the complement system in the pathogenesis of AD [20], [21] and recent proteomic studies have implicated complement proteins in the CSF, including C3a both as biomarkers of established AD [22] as well as predictors of conversion to AD in MCI subjects [23]. Fibrinogen is yet another candidate biomarker common to findings in the current report and a recent proteomic analysis in CSF that identified biomarkers discriminating AD from control samples [24]. It must be noted that very few studies have directly addressed the associations between peripheral concentrations of complement modulating proteins and their levels in the central nervous system. This is an important consideration in the interpretation of blood biomarker studies and their relevance to brain pathology in AD. We have recently attempted to address this question and reported that the plasma concentration of clusterin, a known complement modulator is significantly associated with its expression in brain regions vulnerable to AD pathology [25]. A significant association between γ-fibrinogen and brain volume observed in the current report is also interesting in the light of data demonstrating an increased risk of dementia in subjects with elevated plasma levels of fibrinogen [26].

Our present study suggesting that complement regulators and complement-related proteins are candidate biomarkers of AD also extends findings from our previous proteomic analysis of plasma implicating complement factor-H (CFH) as an AD-specific plasma biomarker [4], [27].

A limitation of the present study that must be acknowledged is its cross-sectional design. Therefore, while our results strongly suggest that we have identified a panel of biomarkers that reflect current disease status by their association with *in vivo* disease pathology, we have not yet extended these findings to examine the utility of these proteins in measuring disease progression. However, our findings merit independent confirmation by other groups and if replicated, are likely to be rapidly extended to longitudinal studies that examine their utility as markers of disease progression or treatment response in clinical trials. It must also be noted that the use of MRI-derived brain volume estimates in this and the majority of other AD biomarker studies may ignore the significant contribution of ischemic microvasular pathology to AD pathogenesis. This issue merits consideration in the interpretation of these studies, especially because of the paucity of reliable imaging biomarkers of microvascular brain injury [28].

In summary, we combined a proteomic and neuroimaging approach to the discovery of biologically relevant biomarkers in AD. Variation in just five plasma proteins, together with age and sex accounts for more than a third of the variance in brain volume suggesting that these proteins are likely to be strong predictors of pathology *in vivo*. We therefore suggest that plasma markers have the potential for future use in large scale community based settings – either in clinical practice or research. Furthermore, these findings add weight to the growing evidence implicating the complement and coagulation pathways in AD pathogenesis.

## Supporting Information

Table S1 **Details of reagents used in Western Blot assays.**
(DOC)

Table S2 **Details of reagents used in ELISA assays.**
(DOC)
