# Supplementary material for: Plasma Biomarkers of Brain Atrophy in Alzheimer's Disease
Source: PLoS One. 2011 Dec 21;6(12):e28527. doi: 10.1371/journal.pone.0028527 (PMC3244409; doi:10.1371/journal.pone.0028527)
Supplement: Table S1 — Details of reagents used in Western Blot assays. (DOC) [file pone.0028527.s001.doc]

**Supporting information**

**Table S1**

| ***Target*** | ***Primary antibody*** | ***Dilution*** | ***Secondary antibody*** | ***Dilution*** |
| --- | --- | --- | --- | --- |
| **Complement factor I** | Human Factor I, A313, Quidel Corporation | 1/20,000 | Alexa Fluor 680 anti-goat IgG A21084, Invitrogen | 1/20,000 |
| **γ-fibrinogen** | Fibrinogen γ (C-20), sc-18032, Santa Cruz Biotechnology | 1/500 | Alexa Fluor 680 anti-goat IgG A21084, Invitrogen | 1/5000 |
| **Serum amyloid precursor (SAP)** | Serum amyloid P, ab40882, Abcam | 1/1000 | Alexa Fluor 680 anti-rabbit IgG A21076, Invitrogen | 1/5000 |
