# Supplementary material for: Plasma Biomarkers of Brain Atrophy in Alzheimer's Disease
Source: PLoS One. 2011 Dec 21;6(12):e28527. doi: 10.1371/journal.pone.0028527 (PMC3244409; doi:10.1371/journal.pone.0028527)
Supplement: Table S2 — Details of reagents used in ELISA assays. (DOC) [file pone.0028527.s002.doc]

**Table S2**

| ***Target*** | ***ELISA kit*** | ***Plasma dilution*** |
| --- | --- | --- |
| **α-1-microglobulin** | α_1_-microglobulin ELISA, K6710, Immunodiagnostik | 1/500 |
| **C3** | Human C3 ELISA, EC2101-1, Gentaur | 1/800 |
| **C3a** | Human C3a ELISA, 550499, BD Biosciences | 1/500 |
|  |  |  |
